# Acute pancreatitis in pregnancy: meta-analysis of maternal and fetal outcomes

**DOI:** 10.1093/bjs/znab221

**Published:** 2021-06-28

**Authors:** D Ll Hughes, A Hughes, P B White, M A Silva

**Affiliations:** Department of Oncology, University of Oxford, Oxford, UK; Department of Hepatobiliary and Pancreatic Surgery, Oxford University Hospitals NHS, Oxford, UK; Cardiff University Medical School, Cardiff, UK; Department of Obstetrics and Gynaecology, University Hospital of Wales, Cardiff, UK; Department of Hepatobiliary and Pancreatic Surgery, Oxford University Hospitals NHS, Oxford, UK

## Abstract

The impact of acute pancreatitis in pregnancy on maternal and fetal outcomes is unclear. Historical case series document very poor outcomes, with maternal mortality rates of 20 per cent and fetal loss of 50 per cent. However, this is based on outdated clinical practice. This meta-analysis quantified both maternal and fetal outcomes following acute pancreatitis in pregnancy based on the concurrent literature. Maternal and fetal outcomes after acute pancreatitis in pregnancy have improved with advances in the management of pancreatitis.

## Introduction

Acute pancreatitis in pregnancy is uncommon, with an estimated incidence rate of between 1 in 1000 and 1 in 10 000 pregnancies^1^^,^^2^. One aspect that remains unclear is the impact of acute pancreatitis in pregnancy on maternal and fetal outcomes. Historical case series reported very poor outcomes, with maternal mortality rates of 20 per cent and fetal loss of 50 per cent^3^^,^^4^. However, these studies are of an era that is not representative of current clinical practice. There is a clear need to define recent outcomes of acute pancreatitis in pregnancy to create risk stratification criteria with which to identify patients most at risk of adverse outcomes. This meta-analysis aimed to quantify maternal and fetal outcomes in acute pancreatitis in pregnancy.

## Methods

The systematic review and meta-analysis were conducted according to PRISMA guidance^5^. A search of four online databases (PubMed, Web of Science, MEDLINE, and Embase) was undertaken. The following search criteria were used to identify the patient-specific cohort: pancreatitis (combined with acute, mild, moderate and severe) and pregnancy.

Two authors independently screened titles and abstracts from the final search against predefined inclusion criteria. The inclusion criteria consisted of studies that described the clinical features of acute pancreatitis in pregnancy. Studies were required to include specific (fetal and/or maternal) outcome measures following acute pancreatitis in pregnancy.

Exclusion criteria comprised case series with less than five patients and non-English language articles. Articles published before 2010 were excluded to determine mortality trends based on studies reflective of current clinical practice.

Data extraction was performed independently by three authors using a predesigned data collection tool. Disagreements were resolved following discussion until consensus was achieved.

### Outcomes of interest

The primary outcome of this meta-analysis was the prevalence of maternal and fetal mortality following acute pancreatitis in pregnancy. The secondary outcome measure aimed to investigate the impact of the timing of onset of pancreatitis in relation to the trimesters of pregnancy on maternal and fetal outcomes.

### Statistical analysis

All statistical analysis was performed using R software version 3.6.3 (R Foundation for Statistical Computing, Vienna, Austria). Each article was assessed formally for methodological quality and risk of bias according to the methodological index for non-randomized studies (MINORS) criteria^6^.

A meta-analysis of outcomes was carried out using a random-effects model incorporating the DerSimonian–Laird method. Results were visualized through forest plots. *I*^2^ values were calculated to assess the degree of heterogeneity among the included studies.

## Results

A total of 8995 patients with acute pancreatitis in pregnancy across 23 studies were included in the meta-analysis (*Table S1*). Included articles were of low methodological quality based on the MINORS criteria (*Table S2*). The mean age at onset of acute pancreatitis in pregnancy was 28.5 years. Pancreatitis predominantly presented in the third trimester of pregnancy (548 patients, 64.9 per cent). Pancreatitis secondary to cholelithiasis was the most frequent cause of pancreatitis, followed by hypertriglyceridaemia (*Table S3*). The severity of the pancreatitis was noted; of 823 patients, over half (467 patients) developed a mild episode of acute pancreatitis. Approximately one-third of patients (249) developed severe pancreatitis (*Table S4*).

**Fig. 1:**
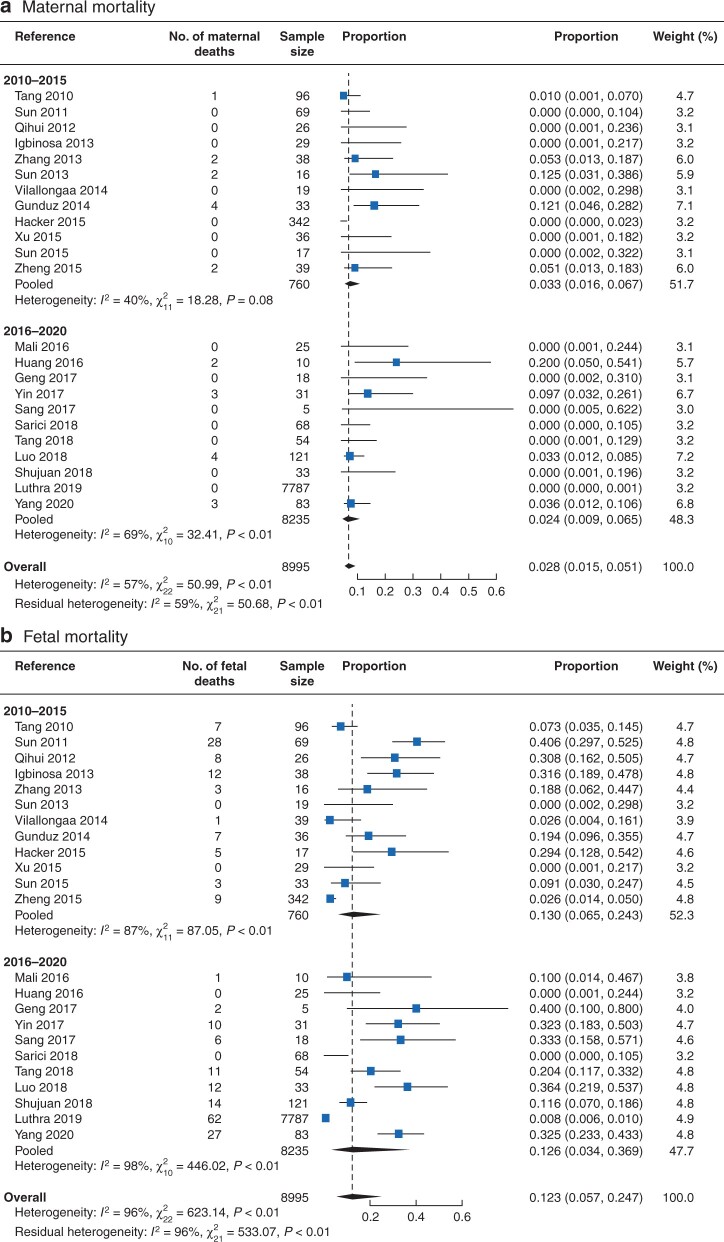
Meta-analysis of pooled maternal and fetal mortality rates **a** Maternal and **b** fetal mortality. A random-effects model was used for meta-analysis. Proportions are shown with 95 per cent confidence intervals.

Maternal and fetal mortality rates for acute pancreatitis in pregnancy are shown in *Fig. 1* and *Table S5*. Meta-analyses of pooled study data demonstrated that the maternal mortality rate following acute pancreatitis in pregnancy was 2.8 (95 per cent c.i. 1.5 to 5.1) per cent (*Fig. 1a*). The pooled fetal mortality rate secondary to acute pancreatitis in pregnancy was 12.3 (5.7 to 24.7) per cent (*Fig. 1b*). Survival trends over time showed an improvement in maternal outcome (mortality rate 2.4 per cent in 2016–2020, reduced from 3.3 per cent in 2010–2015). For fetal outcomes, a much smaller reduction in the mortality rate was observed (12.6 from 13.0 per cent).

Patients were stratified by trimester of onset of acute pancreatitis to determine the impact of acute pancreatitis and gestational age on maternal and fetal outcomes. The timing of onset of acute pancreatitis and mortality outcomes were recorded for 696 patients. Meta-analysis of proportions showed that the pooled rate for maternal death was highest during the first trimester at 12.7 per cent, compared with 7.9 and 6.4 per cent in the second and third trimesters respectively (*Table 1*). Fetal death mirrored this trend; the highest rate of death was in the first trimester (20.9 per cent). Intrauterine fetal death occurred most frequently during the third trimester (8.8 per cent), whereas stillbirth rates were highest during the second trimester (6.2 per cent).

**Table 1:** Maternal and fetal outcomes stratified by trimester of onset of acute pancreatitis

	1st trimester	2nd trimester	3rd trimester
	(*n* = 57)	(*n* = 163)	(*n* = 476)
Maternal death (%)	12.7 (5.6, 26.1)	7.9 (3.1, 18.8)	6.4 (3.7, 10.8)
Fetal death (%)	20.9 (8.0, 44.7)	12.4 (4.4, 30.2)	12.0 (7.0, 19.9)
Intrauterine death (%)	–	7.7 (3.1, 17.8)	8.8 (4.8, 15.5)
Stillbirth (%)	–	6.2 (2.9, 12.9)	4.4 (2.4, 7.9)

Values in parentheses are 95 per cent confidence intervals.

## Discussion

The mortality rate for pregnant women with acute pancreatitis in pregnancy is comparable to the rate described in the general patient population with acute pancreatitis; pregnant women are not at a higher risk of death during the disease course. Patients should be counselled regarding the rates of fetal death per trimester and the increased risk of stillbirth with advancing gestational age.

This meta-analysis has demonstrated improvement in maternal and fetal mortality rates following acute pancreatitis in pregnancy compared with historical data. The overall maternal mortality rate associated with acute pancreatitis during pregnancy was comparable to that of the general population^7^^,^^8^. Fu and colleagues^8^ reviewed 2248 patients with pancreatitis and calculated a 3.8 per cent overall mortality rate. In the present analysis, the fetal mortality rate was highest in the first trimester (20.9 per cent). A possible reason for this is that fetal viability is currently defined at 24 weeks’ gestation. Acute pancreatitis in pregnancy in the first trimester requires a greater time period of growth/survival for fetuses to achieve the viable age, making them vulnerable to an adverse outcome^9^. However, caution is required in interpreting these data. Although the mortality rate may appear high, it is important to highlight that miscarriage in general in the first trimester is common. Current evidence suggests that the present miscarriage rate during the first trimester is approximately 20 per cent^10^^,^^11^. It is not therefore possible to determine whether these patients would have had a miscarriage regardless of developing acute pancreatitis in pregnancy. However, it would seem from this meta-analysis that the stillbirth rate during acute pancreatitis in pregnancy is much higher than that among the general population of pregnant women (4.4–6.2 and 0.5 per cent respectively)^12^. It would appear that the fetal death rate associated with acute pancreatitis in pregnancy is among the highest of fetal mortality rates for surgical emergencies that may occur during pregnancy; rates for other pathologies include 1.6 per cent for simple appendicitis, 7 per cent for non-operative management of cholecystitis, and 17 per cent for small bowel obstruction^13–15^.

Some limitations of this analysis include variation in nomenclature of obstetrics management. ‘Termination’ was used to describe the process of medically induced end of fetal life in some studies, whereas in others it was used to describe termination of pregnancy, that is induction of labour. In such circumstances, a subgroup analysis of fetal death by medical termination was not possible. Acute pancreatitis in pregnancy is uncommon and the included studies contained relatively small cohorts of patients. Missing data and a lack of individual-patient data precluded subgroup analyses, such as the incidence of severe acute pancreatitis per trimester.

*Disclosure*. The authors declare no conflict of interest.

## Supplementary material

Supplementary material is available at *BJS* online.

## Supplementary Material

znab221_Supplementary_Data
