# Supplementary material for: Acute pancreatitis in pregnancy: meta-analysis of maternal and fetal outcomes
Source: Br J Surg. 2021 Jun 28;109(1):12–4. doi: 10.1093/bjs/znab221 (PMC10364714; doi:10.1093/bjs/znab221)
Supplement: znab221_Supplementary_Data [file znab221_supplementary_data.zip › Supplementary_information.docx]

Supplementary information

**Supplementary Table 1.** Study characteristics

| **Author** | **Country** | **Number of patients with AP** | **Maternal age (mean)** | **GA at onset of APIP (mean)** | **Disease onset per Trimester** | | |
| --- | --- | --- | --- | --- | --- | --- | --- |
|  |  |  |  |  | **First Trimester** | **Second Trimester** | **Third Trimester** |
| Fan 2018 | China | 33 | 29 | NR | 1 | 6 | 26 |
| Geng 2017 | China | 18 | 28.9 | NR | - | - | - |
| Gündüz 2014 | Turkey | 33 | 34.8 | 25.7 | 3 | 21 | 9 |
| Hacker 2015 | USA | 342 | <35 285 | NR | - | - | - |
| Huang 2016 | China | 10 | 29.1 | 34.6 | 0 | 1 | 9 |
| Igbinosa 2013 | USA | 29 | 28.7 | NR | 4 | 7 | 14 |
| Luo 2018 | China | 121 | 27.8 | 29.6 | 1 | 42 | 71 |
| Luthra 2019 | USA | 7787 | 26.3 | NR |  |  |  |
| Mali 2016 | USA | 25 | 25.7 | 24.4 | 5 | 5 | 15 |
| Sun 2011 | China | 69 | 29 | 28.9 | 5 | 29 | 35 |
| Sarıcı 2018 | Turkey | 68 | 26 | NR | 24 | 6 | 38 |
| Sang 2017 | China | 5 | 32 | 31 |  |  | 5 |
| Qihui 2012 | China | 26 | 28.35 | 25.7 | - | - | - |
| Zheng 2015 | China | 39 | 27.6 | all>28 weeks | 0 | 0 | 39 |
| Zhang 2013 | China | 38 | 28 | 32 | 2 | 6 | 30 |
| Yin 2017 | China | 31 | 29.4 | 31 | 1 | 7 | 23 |
| Yang 2020 | China | 83 | 29 | NR | 1 | 11 | 71 |
| Xu 2015 | China | 36 | NR | NR | 2 | 9 | 22 |
| Vilallongaa 2014 | Spain | 19 | 30.9 | NR |  |  | 10 |
| Tang 2010 | USA | 96 | 26 | NR | 27 | 26 | 43 |
| Tang 2018 | China | 54 | 27.2 | 29.1 | 2 | 18 | 32 |
| Sun 2013 | China | 16 | 31.5 | 36.4 | 0 | 0 | 16 |
| Sun 2015 ^43^ | China | 17 | 28 | 34.8 | 0 | 0 | 17 |

**Supplementary Table 2.** Methodological index for non-randomised studies (MINORS) assessment of included studies

| Study | Clearly Stated Aim | Inclusion of Consecutive Patients | Prospective Data Collection | End points Appropriate to Study Aim | Unbiases Assessment of Study Endpoint | Follow-Up Period Appropriate to Study Aim | <5% Lost  to Follow-Up | Prospective Calculation of Study Size | Total |
| --- | --- | --- | --- | --- | --- | --- | --- | --- | --- |
| Chen 1995 | 2 | 2 | 0 | 2 | 0 | 2 | 2 | 0 | 10 |
| Ramin 1995 | 2 | 2 | 0 | 0 | 0 | 0 | 0 | 0 | 4 |
| Hernandez 2007 | 2 | 2 | 0 | 2 | 0 | 0 | 1 | 0 | 7 |
| Crisan 2008 | 2 | 2 | 0 | 1 | 0 | 0 | 2 | 0 | 7 |
| Tang 2010 | 1 | 2 | 0 | 1 | 0 | 1 | 0 | 0 | 5 |
| Sun 2011 | 2 | 2 | 0 | 2 | 0 | 1 | 2 | 0 | 9 |
| Qihui 2012 | 2 | 2 | 0 | 2 | 0 | 1 | 2 | 0 | 9 |
| Igbinosa 2013 | 2 | 2 | 0 | 2 | 0 | 2 | 2 | 0 | 10 |
| Sun 2013 | 2 | 1 | 0 | 2 | 0 | 0 | 1 | 0 | 6 |
| Vilallongaa 2014 | 2 | 2 | 0 | 2 | 0 | 1 | 2 | 0 | 9 |
| Gunduz 2014 | 2 | 2 | 0 | 2 | 0 | 0 | 2 | 0 | 8 |
| Hacker 2015 | 2 | 2 | 0 | 2 | 0 | 0 | 2 | 2 | 10 |
| Xu 2015 | 2 | 2 | 0 | 2 | 0 | 1 | 2 | 0 | 9 |
| Sun 2015 | 2 | 2 | 0 | 2 | 0 | 2 | 2 | 0 | 10 |
| Zheng, 2015 | 2 | 2 | 0 | 1 | 0 | 1 | 2 | 0 | 8 |
| Mali 2016 | 2 | 2 | 0 | 2 | 0 | 0 | 2 | 0 | 8 |
| Huang 2016 | 2 | 2 | 0 | 2 | 0 | 1 | 2 | 0 | 9 |
| Geng 2017 | 2 | 2 | 0 | 2 | 0 | 0 | 2 | 1 | 9 |
| Yin 2017 | 1 | 2 | 0 | 2 | 0 | 1 | 2 | 0 | 8 |
| Sang 2017 | 2 | 2 | 0 | 2 | 0 | 2 | 2 | 0 | 10 |
| Sarıcı 2018 | 2 | 2 | 0 | 2 | 0 | 2 | 2 | 0 | 10 |
| Tang 2018 | 2 | 1 | 0 | 2 | 0 | 1 | 2 | 0 | 8 |
| Luo 2018 | 2 | 2 | 0 | 2 | 0 | 0 | 2 | 0 | 8 |
| Shujuan 2018 | 2 | 2 | 0 | 2 | 0 | 0 | 2 | 0 | 8 |
| Luthra 2019 | 2 | 2 | 0 | 1 | 0 | 1 | 2 | 2 | 10 |
| Yang 2020 | 2 | 2 | 0 | 2 | 0 | 1 | 2 | 0 | 9 |

**Supplementary Table 3.** Studies reporting aetiologies of acute pancreatitis

| **Author** | **Aetiology of pancreatitis**^*^**^^^** | | | | |
| --- | --- | --- | --- | --- | --- |
|  | **Biliary** | **Alcohol** | **Idiopathic** | **Hypertriglyceridemia** | **Other** |
| Fan 2018 | 13 | 0 | 4 | 14 | 0 |
| Geng 2017 | 11 | 0 | 2 | 8 | 0 |
| Gündüz 2014 | 18 | 0 | 4 | 11 | 3 |
| Hacker 2015 | NR | NR | NR | NR | NR |
| Huang 2016 | 0 | 0 | 0 | 10 | 0 |
| Igbinosa 2013 | 21 | 1 | 7 | 0 | 0 |
| Luo 2018 | 44 | 0 | 32 | 39 | 60 |
| Luthra 2019 | NR | NR | NR | NR | 0 |
| Mali 2016 | 14 | 4 | 1 |  | 0 |
| Sun 2011 | 29 | 5 |  | 14 | 0 |
| Sarıcı 2018 | 68 | 0 | 0 | 0 | 0 |
| Sang 2017 | 2 |  |  | 3 | 0 |
| Qihui 2012 | NR | NR | NR | NR | NR |
| Zheng 2015 | NR | NR | NR | NR | NR |
| Zhang 2013 | 11 | NR | NR | 5 | 0 |
| Yin 2017 | 5 | 0 | 14 | 12 | 0 |
| Yang 2020 | 20 | 0 |  | 41 | 22 |
| Xu 2015 | 7 | 0 | 5 | 14 | 10 |
| Vilallongaa 2014 | 17 | 0 | 1 | 1 | 0 |
| Tang 2010 | 89 | 0 | 0 | 0 | 7 |
| Tang 2018 | 14 | 0 | 0 | 22 | 18 |
| Sun 2013 | 11 | 0 | 1 | 4 | 0 |
| Sun 2015 ^43^ | 7 | 1 | 0 | 8 | 4 |

**Supplementary Table 4.** Studies reporting disease severity

| **Author** | **Country** | **Severity assessment criteria** | **Pancreatitis severity** | | | |
| --- | --- | --- | --- | --- | --- | --- |
|  |  |  | **MAP** | **MP** | **MSAP** | **SAP** |
| Fan 2018 | China | Other^&^ | 22 | 5 | 6 | - |
| Geng 2017 | China | Atlanta | - | - | - | 18 |
| Gündüz 2014 | Turkey | Other^&^ | 15 | - | - | 18 |
| Hacker 2015 | USA | NS | - | - | - | - |
| Huang 2016 | China | Atlanta | 1 | 5 | 4 | - |
| Igbinosa 2013 | USA | Atlanta | - | - | - | - |
| Luo 2018 | China | Atlanta | 59 |  | 44 | 18 |
| Luthra 2019 | USA | NS | - | - | - | - |
| Mali 2016 | USA | Other^&^ | - | - | - | - |
| Sun 2011 | China | APACHE II score | 51 | - | - | 18 |
| Sarıcı 2018 | Turkey | Atlanta | 61 | 7 | - | - |
| Sang 2017 | China | Imaging^$^ | 3 |  | 1 | 1 |
| Qihui 2012 | China | NS | 22 | - | - | 4 |
| Zheng 2015 | China | Other^&^ | - | - | - | 39 |
| Zhang 2013 | China | APACHE II score | 26 | - | - | 12 |
| Yin 2017 | China | Atlanta | 15 | 8 | - | 8 |
| Yang 2020 | China | Imaging^$^ | - | - | - | 83 |
| Xu 2015 | China | Atlanta | 19 | - | - | 17 |
| Vilallongaa 2014 | Spain | Other^&^ | 19 | - | - | - |
| Tang 2010 | USA | Not specified | 97 | - | - | 6 |
| Tang 2018 | China | APACHE II score | 24 |  | 24 | 7 |
| Sun 2013 | China | Other^&^ | 9 | - | - | 7 |
| Sun 2015 ^43^ | China | Atlanta | 3 |  | 3 | 11 |

GA - Gestational age, APIP - Acute Pancreatitis in Pregnancy, MAP - Mild Acute Pancreatitis, M – Moderate Acute Pancreatitis, MSAP – Moderate-Severe Pancreatitis, SAP – Severe Acute Pancreatitis, *Aetiological cause not recorded for each patient within all studies ^ Multiple aetiological causes recorded for some patient within the included studies, $ Imaging criteria includes CT severity index and Balthazar scoring, & Other included country specific pancreatitis diagnosis guidelines

**Supplementary Table 5**. Meta-regression for maternal and foetal mortality

| Foetal mortality | | | |
| --- | --- | --- | --- |
| Variable for meta regression | Estimate | 95% CI | p-value |
| Mild Pancreatitis | -0.0189 | - 0.0380 - 0.0002 | 0.0527 |
| Moderate Pancreatitis | -0.2433 | -0.6997 - 0.2132 | 0.2962 |
| Moderate-Severe Pancreatitis | -0.0083 | -0.0426 - 0.0261 | 0.6380 |
| Severe Pancreatitis | 0.0185 | -0.0186 - 0.0557 | 0.3288 |
| Onset during first trimester | -0.0384 | -0.0977 - 0.0210 | 0.2050 |
| Onset during second trimester | -0.0384 | -0.0977 - 0.0210 | 0.2050 |
| Onset during third trimester | -0.0045 | -0.0273 - 0.0183 | 0.7003 |
| Acute fluid collection | -0.0006 | -0.0344 - 0.0332 | 0.9718 |
| Pseudocyst | -0.1126 | -0.2576 - 0.0324 | 0.1279 |
| DIC | 0.2119 | -0.2926 - 0.7164 | 0.4104 |
| Respiratory failure | -0.0018 | -0.0283 - 0.0248 | 0.8968 |
| Renal failure | -0.0772 | -0.1367 - 0.0177 | 0.0109 |
| Maternal mortality | | | |
| Variable for meta regression | Estimate | 95% CI | p-value |
| Mild Pancreatitis | -0.0165 | -0.0392 - 0.0062 | 0.1543 |
| Moderate Pancreatitis | -0.2990 | -0.6783 - 0.0803 | 0.1224 |
| Moderate-Severe Pancreatitis | -0.0156 | -0.0416 - 0.0104 | 0.2390 |
| Severe Pancreatitis | -0.0028 | -0.0250 - 0.0193 | 0.8023 |
| Onset during first trimester | -0.0491 | -0.1109 - 0.0128 | 0.1198 |
| Onset during second trimester | -0.0491 | -0.1109 - 0.0128 | 0.1198 |
| Onset during third trimester | -0.0121 | -0.0257 - 0.0015 | 0.0823 |
| Acute fluid collection | 0.0068 | -0.0319 - 0.0454 | 0.7318 |
| Pseudocyst | -0.1319 | -0.4637 - 0.1999 | 0.4360 |
| DIC | 0.3413 | -0.3947 - 1.0773 | 0.3634 |
| Respiratory failure | 0.0110 | -0.0165 - 0.0385 | 0.4325 |
| Renal failure | -0.0336 | -0.2175 - 0.1503 | 0.7205 |
